# Deep Learning-Enabled Integration of Histology and Transcriptomics for Tissue Spatial Profile Analysis

**DOI:** 10.34133/research.0568

**Published:** 2025-01-17

**Authors:** Yongxin Ge, Jiake Leng, Ziyang Tang, Kanran Wang, Kaicheng U, Sophia Meixuan Zhang, Sen Han, Yiyan Zhang, Jinxi Xiang, Sen Yang, Xiang Liu, Yi Song, Xiyue Wang, Yuchen Li, Junhan Zhao

**Affiliations:** ^1^ School of Big Data and Software Engineering, Chongqing University, Chongqing, China.; ^2^ Department of Computer and Information Technology, Purdue University, West Lafayette, IN, USA.; ^3^ Radiation Oncology Center, Chongqing University Cancer Hospital, Chongqing, China.; ^4^ Chongqing Key Laboratory of Translational Research for Cancer Metastasis and Individualized Treatment, Chongqing University Cancer Hospital, Chongqing, China.; ^5^ Tri-Institutional Computational Biology & Medicine, Weill Cornell Medicine, New York, NY, USA.; ^6^ Department of Computational Oncology, Memorial Sloan Kettering Cancer Center, New York, NY, USA.; ^7^ College of Agriculture and Life Sciences, Cornell University, Ithaca, NY, USA.; ^8^ Harvard College, Harvard University, Cambridge, MA, USA.; ^9^ Division of Genetics, Brigham and Women’s Hospital and Harvard Medical School, Boston, MA, USA.; ^10^ Department of Biostatistics, University of North Carolina at Chapel Hill, Chapel Hill, NC, USA.; ^11^ Department of Genetics, University of North Carolina at Chapel Hill, Chapel Hill, NC, USA.; ^12^ Department of Biostatistics, Harvard T.H. Chan School of Public Health, Boston, MA, USA.; ^13^ Department of Radiation Oncology, Stanford University School of Medicine, Stanford, CA, USA.; ^14^ Department of Biostatistics and Health Data Science, Indiana University School of Medicine, Indianapolis, IN, USA.; ^15^ Department of Neurosurgery, Chongqing University Three Gorges Hospital, Chongqing, China.; ^16^ Department of Biomedical Informatics, Harvard Medical School, Boston, MA, USA.

## Abstract

Spatially resolved transcriptomics enable comprehensive measurement of gene expression at subcellular resolution while preserving the spatial context of the tissue microenvironment. While deep learning has shown promise in analyzing SCST datasets, most efforts have focused on sequence data and spatial localization, with limited emphasis on leveraging rich histopathological insights from staining images. We introduce GIST, a deep learning-enabled gene expression and histology integration for spatial cellular profiling. GIST employs histopathology foundation models pretrained on millions of histology images to enhance feature extraction and a hybrid graph transformer model to integrate them with transcriptome features. Validated with datasets from human lung, breast, and colorectal cancers, GIST effectively reveals spatial domains and substantially improves the accuracy of segmenting the microenvironment after denoising transcriptomics data. This enhancement enables more accurate gene expression analysis and aids in identifying prognostic marker genes, outperforming state-of-the-art deep learning methods with a total improvement of up to 49.72%. GIST provides a generalizable framework for integrating histology with spatial transcriptome analysis, revealing novel insights into spatial organization and functional dynamics.

## Introduction

Advances in spatial molecular imaging have enabled the examination of spatial transcriptional profiles within complex tissues at a subcellular resolution [1–3]. Exploring the spatial coordinates and the transcriptional profiles of individual cells within tissue mircroenvironment deepensour understanding of the spatial diversity in cellular interactions. Commercially available technologies for single-cell spatial profiling, such as the NanoString CosMx Spatial Molecular Imager (SMI) [4] and Vizgen MERSCOPE/MERFISH platforms [4,5], have demonstrated promising results in providing transcriptional profiles, cell locations and boundaries, and multichannel imaging modalities. For example, the NanoString CosMx platform can simultaneously interrogate up to 1,000 genes and analyze 100,000 to 600,000 cells per slide, surpassing the prevailing single-cell omics methodologies. These emerging single-cell spatial transcriptomics (SCST) platforms, along with accurate and timely histopathological assessments [6], are catalyzing a paradigm shift in biomedical research, advancing our understanding of complex tissue architecture both spatially and functionally and disease mechanisms with unprecedented resolution [7,8].

Refining spatial gene expression data remains a substantial challenge. Spatial transcriptomics (ST) profiles are affected by issues such as missing values [9], data sparsity [10], low coverage [2], and noise [11], which complicate effective biological exploration, especially creating precise training dataset for artificial neural networks [12]. Meanwhile, multiplex immunofluorescence images within single-cell spatial data capture high-resolution, detailed features observed in tissue samples, including cell types, cellular compartment morphologies, and spatial cell distributions. The integration of such imaging attributes with transcriptomic data holds promise for mitigating challenges arising from missing values and data noise. Given that spatial relationships between individual cells and their neighboring counterparts can naturally be represented through a spatial adjacency graph, making graph-based artificial intelligence an intuitive approach for spatial data modeling. Notably, graph-based models augmented with attention mechanisms, such as the GAT and graph transformer models [13,14], have shown promising advancements and enhanced investigation outcomes.

Accurate identification of spatial domains of tissues is critical but challenging for understanding distinct anatomical and functional regions. Current methods using SCST data focus on revealing spatial clusters, such as the integrated tool Seurat [15] and Scanpy [16]. These clustering techniques were originally proposed for processing nonspatial single-cell RNA-sequencing data. Therefore, only gene expression data are used as input. Researchers have attempted to integrate gene expression data with basic spatial and cellular information to refine the identification of spatial domains. StLearn [17] exploits both gene expression profiles and features extracted from tissue images. BayesSpace [18] employs a Bayesian statistical framework, which analyzes gene expression matrix and spatial proximity information. Additionally, SpaGCN [19] uses a graph convolutional network to identify spatial domains by constructing a spatial graph of gene expression based on the geospatial information in the images. STAGATE [20] leverages the graph attention network (GAT) [21] to dynamically consider nearby gene expressions. By integrating morphological and spatially resolved transcription data, MUSE [22] uses a multimodal structured embedding approach to find any tissue subgroups that are missed by multiple modes and compensate for pattern-specific noise. PROST [23] has optimized the integration of spatial information and gene expression profiles through 2 key modules, PROST Index (PI) and PROST Neural Network (PNN). CellCharter [24] leverages variational autoencoders (VAE) to enhance the merging of cellular characterization with histopathology. Despite the demonstrated efficacy of these methods, the potential to harness cellular morphological information embedded within spatial imaging profiles, extending beyond analogous cell localization, remains underutilized.

 Many advanced deep learning-based methods were proposed to better extract the image features. STACI [25] analyzes spatial transcriptomics gene data and chromatin imaging data by employing overparameterized graph-based autoencoders. To reduce the interference of noise in ST data, TIST [26] extracts complementary cellular phenotypic information in high-resolution histopathology images by comprehensively analyzing transcriptomic data and images. Leng et al. [27] proposed a label-efficient approach that leverages curriculum learning and confidence learning to detect noise for the analysis of ST data. To decipher intercellular communication within spatial transcriptomics graphs, BLEEP [28] constructs paired images and expression profiles simultaneously using contrast learning at micrometer resolution, thus mapping the original dataset to a low-dimensional joint embedding space. TCGN [29] takes advantage of convolutional neural networks (CNNs), the transformer encoder, and graph neural networks (GNNs) as input for histopathological image analysis to process the pathology images in ST data. SiGra [30] utilizes graph transformers to achieve state-of-the-art performance by aggregating morphology features from surrounding cells. However, these methods have not been developed for fully utilizing histology image features for specifically extracting the unique morphological characteristics of single-cell spatial data, instead relying on vision models trained on natural images, which primarily treat histological images as general image data or apply basic image-processing techniques like segmentation. This approach results in a lack of trained histopathological perception and domain-specific intelligence necessary for fully interpreting the images. 

Deep learning-enabled digital pathology has uncovered quantitative morphological signals in histology images that are indicative of diagnostics and prognostic prediction [31–35]. PhaseFIT [36] improves image generation by utilizing a segmentation algorithm that precisely executes image translation while integrating channel-wise and spatial-wise attention to concentrate on the most influential feature maps. The use of self-supervised learning (SSL) to train pathology foundation models [35,37] with millions of histology images has advanced significantly in recent years. CTransPath [38], as a pioneering histology foundation model, employed a CNN and vision transformer [39] hybrid trained on 15.6 million tiles from 32,220 whole-slide images spanning 25 anatomic sites and over 32 cancer subtypes. This model has been independently evaluated for different tasks, such as image retrieval, disease classification, mitosis detection, and lesion segmentation. Afterward, UNI [37], directly training on 100 million tiles using DINOv2 [40] architecture, was successfully validated by 33 pathology analytical tasks. These methods highlight the potential of SSL to enhance visual features without incurring high dataset labeling costs. Similarly, Virchow2 [41] was trained on 3.1 million histopathological whole-slide images using a domain-inspired training approach, functioning as a visual converter with 632 million parameters. Prov-GigaPath [42] was pretrained on 1.3 billion 256 × 256 pathology image tiles derived from 171,189 whole slides. To capture both local and global patterns across entire slices, Prov-GigaPath transformed slides into long strings of visual markers by tiling the images into these markers. UNI, Virchow2, and Prov-GigaPath all utilize the DINOv2 framework, while claiming distinct pretraining strategies tailored to different datasets.

In this paper, we established a novel deep learning framework for multimodal SCST data analysis, named GIST (Gene expression and histology Integration for SpaTial cellular profiling). GIST leverages self-supervised histology image foundation models to extract detailed morphological features of tissues and cells. By integrating multimodal data through hybrid graph encoding, GIST efficiently combines morphological information with transcriptomic data to precisely identify cell types and analyze spatial expression patterns. We showed that GIST effectively denoises ST data and excels in downstream tasks, including spatial domain identification, amplification of specific marker gene detection, and differential expression gene analysis. We validated the generalizable performance of GIST using human lung, breast, and colorectal cancer datasets collected using different ST platforms. GIST outperformed the state-of-the-art deep learning methods and improved the accuracy of segmenting the microenvironment and denoising transcriptomics data by up to 49.72%. GIST potentially serves as a robust framework for integrating histology and spatial gene expression data, offering a scalable approach for analyzing spatial transcriptomic data and understanding complex diseases.

## Results

### Overview of the GIST

**Fig. 1.**
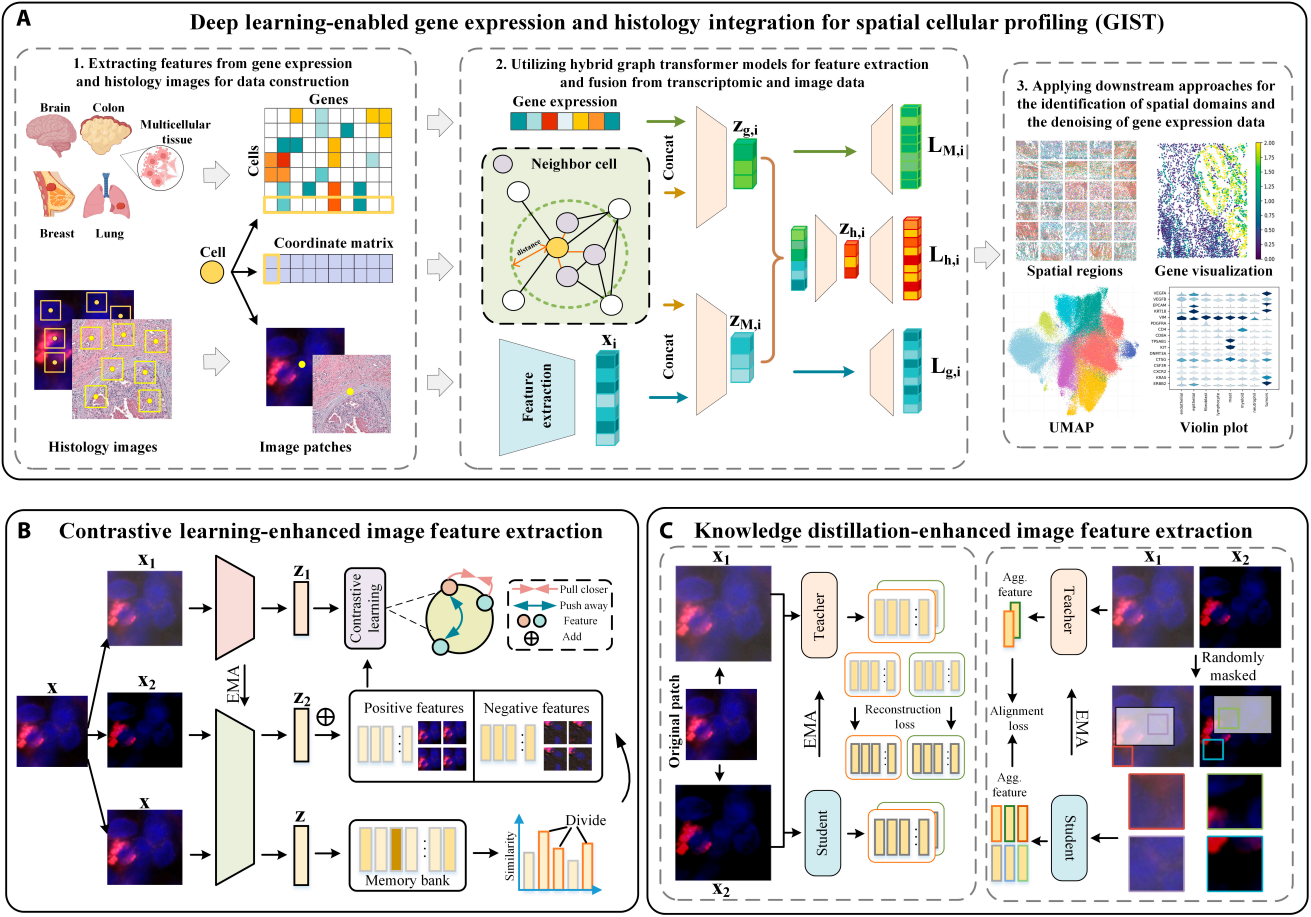
Overview of GIST. (A) Integration of gene expression and histology features for spatial transcriptomics data analysis. GIST leverages a hybrid graph transformer to efficiently fuse feature representations from (1) next-generation sequencing, which identifies cell types and gene spatial locations at various anatomical sites, and (2) morphological features in tissues. This fusion enables downstream tasks such as data denoising, spatial domain identification, and enhanced characterization of gene expression patterns. GIST employs pathology image foundation models pretrained on millions of histological images to extract comprehensive image feature representations. Two predominant foundational model architecture families trained using self-supervised learning strategies are employed. (B) Contrastive learning for image feature extraction. Image patches are augmented to create multiple views, processed through parallel networks, generating feature vectors. These vectors are compared to similar samples from a memory bank, with contrastive learning and EMA refining the representations. (C) Knowledge distillation-enhanced image feature extraction. The enhanced views generated from patches of the original image are utilized to compute the reconstruction loss. Subsequently, the augmented views are randomly masked to compute an alignment loss, which evaluates the feature representation of each masked patch. The graphics of the human organs in (A) were created with BioRender.

We developed a novel GIST method, i.e., a deep learning-enabled gene expression and histology integration for spatial cellular profiling. GIST leverages histopathology image foundation models for extracting image features and employs hybrid graph transformers to fuse features from both transcriptomics and tissue images (Fig. 1). To demonstrate the generalizability of GIST, we applied it to diverse tissue sections, including lung, human breast cancer, and colorectal tissues, achieving notable results in spatial domain identification and differential gene expression analysis.

Our GIST framework consists of 3 main components: (a) feature extraction from both transcriptomic data (gene expression) and histology images, (b) a hybrid graph transformer model for fusing the multimodal features , and (c) downstream approaches for spatial domain identification through denosing gene expression data (Fig. 1A). In the data preprocessing stage, we obtained gene expression profiles and cell spatial location information from spatial transcriptomic data and selected histology images containing cell morphology as multimodal input to GIST. In feature extractor and hybrid graph transformer model stage, we first identified cell positions in larger histological images using the spatial location information of cells and then extracted image features using foundation models from these smaller image patches accordingly. This approach enhances the discriminative power of the learned representations (Fig. 1B and C). The extracted image features and the transcriptome features processed from the gene expression data were then input into hybrid graph transformer model to obtain the final enhanced representations. In downstream analysis stage, we analyzed the transcriptomic alterations within the enhanced datasets generated by GIST, facilitating various forms of downstream tasks.

### Data sources

We validated GIST using spatial transcriptomic data for 3 different anatomic sites, namely, formalin-fixed, paraffin-embedded (FFPE) non-small cell lung cancer (NSCLC) tissue samples obtained by NanoString CosMx SMI [43], FFPE human breast tissue, and fresh-frozen invasive ductal carcinoma breast tissue from BioIVT Asterand obtained by 10x Genomics and FFPE human colorectal cancer tissue from Discovery Life Sciences obtained by 10x Genomics. The NanoString FFPE NSCLC dataset encompasses 8 tissue samples with non-small cell lung cancer (NSCLC). Each NSCLC sample is associated with a range of 20 to 45 high-resolution images. Samples labeled lungs 5-1, 5-2, and 5-3 originate from a single patient, and lungs 9-1 and 9-2 are also from a single patient. The remaining samples are derived from individual patients, each contributing to the dataset’s diversity. The FFPE tissues contain diverse cellular populations, identifying 18 distinct cell types. These classifications are further divided into 8 primary cell types: endothelial, epithelial, fibroblast, lymphocyte, mast, myeloid, neutrophil, and tumor cells. Regarding the human breast cancer datastes, we utilized 2 spatial gene expression datasets from human breast cancer specimens, each processed with different versions of the Space Ranger: Version 1.0 and Version 1.3. The dataset processed with Space Ranger Version 1.0 consists of samples from freshly frozen invasive ductal carcinoma of the mammary tissue. The dataset processed with Space Ranger Version 1.3 is derived from FFPE human breast tissue specimens. For human colorectal cancer, the spatial gene expression dataset was prepared using Space Ranger Version 2.0.1. The examination of hematoxylin and eosin (H&E) images revealed colorectal cancers exhibiting a connective tissue proliferative response.

### GIST deciphers cell types in the single-cell spatial landscapes of lung cancer

To quantitatively evaluate cell type identification in lung cancer, we applied GIST to CosMx SMI dataset on 8 FFPE NSCLC specimens. We benchmarked the clustering accuracy of GIST against 8 state-of-the-art spatial clustering methods using the adjusted rand index (ARI) as the evaluation metric. Compared to other methods, GIST exhibited significantly better ARI indices (Fig. S1A and Table S2). GIST with UNI reached an average ARI of 0.61, which surpasses the performance of state-of-the-art models, CellCharter (ARI = 0.50), followed by SiGra (ARI = 0.47), stlearn (ARI = 0.42), Seurat (ARI = 0.33), Scanpy (ARI = 0.31), BayesSpace (ARI = 0.27), spaGCN (ARI = 0.25), and STAGATE (ARI = 0.22). The ARI distribution across all FFPE NSCLC samples markedly improved with GIST. Notably, GIST’s ability to address an outlier underscores its efficacy in resolving aberrant data points. 

**Fig. 2.**
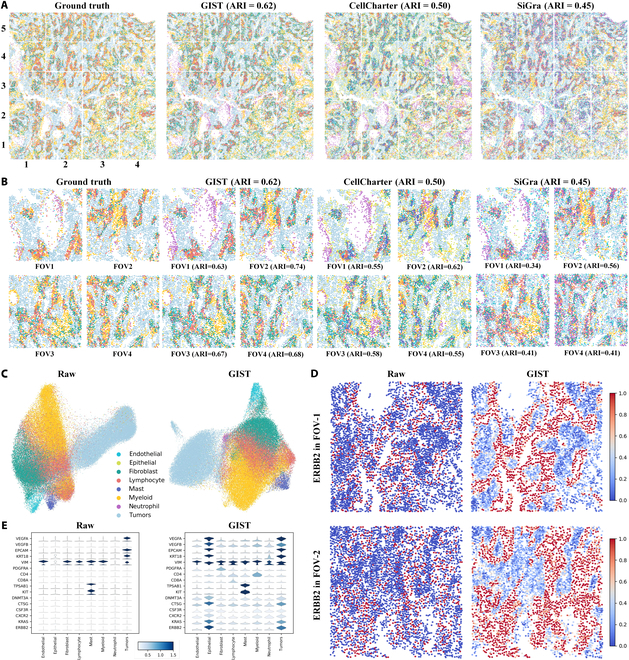
GIST deciphered cell types in the single-cell spatial landscapes of lung cancer. (A) Spatial region comparisons in an FFPE NSCLC sample (lung13) among ground truth, GIST (ARI = 0.62), CellCharter (ARI = 0.50), and SiGra (ARI = 0.45). GIST achieved a closer match to the ground truth in spatial domain recognition. (B) Comparison of tumor-dominated FOVs (FOV1: row 2, column 2; FOV2: row 3, column 2) and multicellular-based FOVs (FOV3: row 4, column 3; FOV4: row 5, column 3) among ground truth, GIST, CellCharter, and SiGra. GIST accurately distinguished both tumor-dominated and multicellular regions, demonstrating its robustness in cell classification. (C) Tumor cell types were well discriminated, with some inter-class segregation and intra-class aggregation observed in myeloid and endothelial groups. (D) Spatial visualization of raw and enhanced ERBB2 gene expression in 2 FOVs (*t* test, *P* = 5.3 × 10^−7^). GIST significantly enhanced the identification of ERBB2 in tumor regions, aiding in the detection of this oncogenic driver in NSCLC. (E) Visualizations show that GIST preserved the original gene expression profiles while highlighting the expression of gene-of-interests (e.g., ERBB2), particularly those associated with tumor growth.

GIST also enhanced the spatial domain detection and clustering results of the CosMx SMI dataset. For example, in the FFPE NSCLC slice lung13, GIST with UNI achieved the highest clustering accuracy of predicting spatial domains among all tested methods in Fig. 2A (ARI = 0.62). The FFPE lung13 sample comprises 77,043 cells and encompasses 960 genes, organized into 20 fields of view (FOVs). Results for other samples are available in the Supplementary Materials (Figs. S5 and S6). Spatial clustering results at FOV level showed that GIST’s predictions matched with the ground truth (Fig. 2B). Across FOV1 and FOV2, characterized by heightened tumor concentrations, GIST accurately discerned these focal areas and effectively delineated adjacent regions intermixed with diverse cellular constituents. Whether encountering tumors juxtaposed with lymphocytes (FOV3) or myeloid cells (FOV4), GIST precisely categorized these compositions, further exemplifying its efficacy in spatial domain analysis. Conversely, Cellcharter misidentified myeloid cells as mast and fibroblast. While Cellcharter performed adequately in tumor-dominated FOVs (FOV2), its accuracy degraded in multicellular-based FOVs (FOV3 and FOV4), indicating a limited capability to distinguish intricate biological scenarios involving the fusion of multiple cell types. SiGra misidentified myeloid cells as neutrophil almost in all FOVs. In contrast, GIST demonstrated proficiency in both scenarios, underscoring its expansive utility in discerning cell types.

### GIST enhances lung cancer-based gene expression of NanoString CosMx SMI

GIST also enhances the detection and characterization of clinically relevant gene markers in downstream analysis by refining data quality and resolution. We applied Uniform Manifold Approximation and Projection (UMAP) to reduce the dimensionality of the original dataset to visualize the clusters of cell types based on the feature similarities corresponding to both the original SCST dataset and the GIST-enhanced dataset (Fig. 2C). The enhanced dataset from GIST revealed a more discernible separation in the reduced-dimensional space. Notably, the enhanced dataset facilitated tumor segmentation and also improved the differentiation of cell types that were previously merged in the original dataset, such as fibroblast and endothelial cells.

Preclinical and clinical studies have identified ERBB2, also commonly known as HER2, as a targetable driver mutation in NSCLC [44]. We visualized the expression of the tumor-specific gene ERBB2 in Fig. 2D and found that its expression was significantly enhanced in the GIST dataset (*t* test, *P* = 5.3 × 10^−7^). GIST enabled more accurate detection of ERBB2 in tumor regions, aiding in the assessment of the functional consequences of the mutation. Comparing cell type-specific gene expression between the original dataset and the GIST-enhanced dataset, GIST amplified the visibility of certain gene-of-interests while maintaining the general expression trends of the original dataset (Fig. 2E). For instance, KRAS, a frequently mutated oncogene in NSCLC, is implicated in predicting clinical outcomes for patients undergoing diverse treatments [45]. Additionally, ERBB2 represents a therapeutic target mutation in NSCLC patients, and ERBB2-directed therapies can be effective in managing disease progression in individuals with metastatic ERBB2-mutated NSCLC [46]. To elucidate the expression profiles of these genes in lung cancer tissues, we visualized the expression patterns of KRAS and ERBB2, revealing high expression in tumor regions. These genes can not be identified in the raw data, demonstrating GIST’s capability to improve gene expression analysis in lung cancer tissues.

### GIST effectively identifies additional prognostic marker genes from differentially expressed genes in human breast cancer

GIST was further evaluated on human breast tissues sampled using BioIVT Asterand. The first FFPE human breast tissue (Space Ranger 1.3.0) contains 2,518 cells and 17,943 genes including four annotation classes : desmoplastic changes, lymphocytes, necrosis and hemorrhage, and tumor. The second human breast cancer (Space Ranger 1.0.0), consists of freshly frozen invasive ductal breast cancer tissue, comprising 3,813 cells and 33,538 gene data points annotated in 3 classes: desmoplastic changes, lymphocytes, and tumors. 

**Fig. 3.**
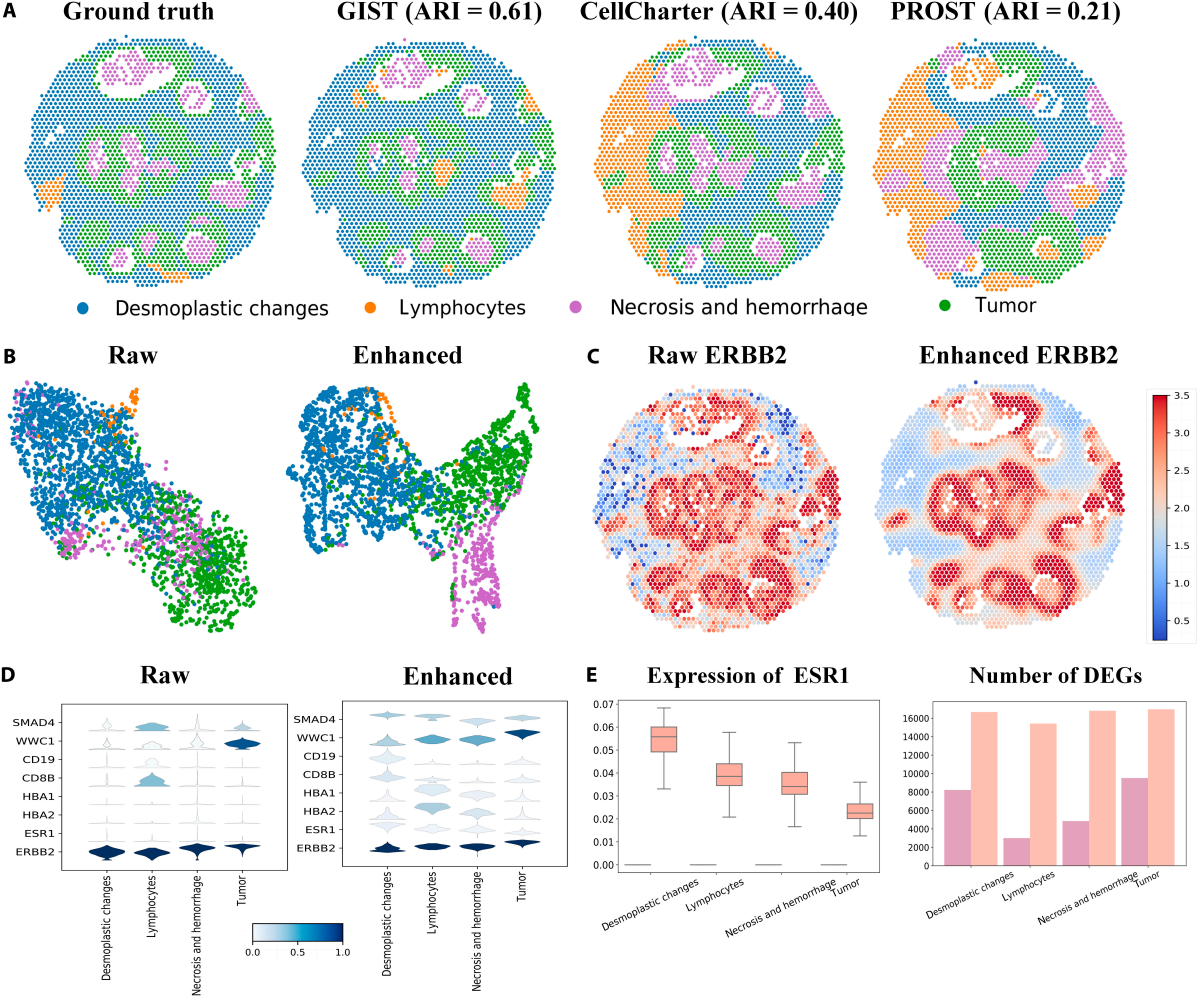
GIST enhanced the identification of marker genes from differentially expressed genes (DEGs) in human breast cancer. (A) Spatial regions predicted by GIST (ARI = 0.61), CellCharter (ARI = 0.40), and PROST (ARI = 0.21) compared to ground truth. GIST predictions exhibit strong alignment with the ground truth, particularly in accurately delineating tumor regions. (B) In the raw UMAP, tumor cells in red dots are scattered, making them difficult to distinguish from other cell types. After enhancement, tumor cells are clustered together, improving their identification and separation. (C) Spatial visualization of raw and enhanced ERBB2 expression in an FFPE human breast sample. The enhanced visualization reduces noise and reveals clearer patterns of ERBB2 expression (*t* test, *P* = 0.00084), demonstrating the effectiveness of the enhancement method. (D) GIST-enhanced gene expression patterns improve the identification of marker genes associated with specific biological processes or cancer states, such as ERBB2. (E) Visualization and comparison of raw (purple) and enhanced (orange) ESR1 expression across different cell clusters highlight expression patterns and the number of DEGs that are not apparent in the raw data but become evident with enhancement.

The spatial regions of human breast cancer (Space Ranger 1.3.0) were depicted in comparison with the ground truth (Fig. 3A). The spatial regions predicted by GIST demonstrated greater accuracy compared to the ground truth than baseline models such as CellCharter and PROST, especially within the tumor region. We further conducted a comparative analysis using both raw and enhanced datasets (Fig. 3B). In the raw dataset, cells associated with tumor types are clustered together with cells indicative of necrosis and hemorrhage. In contrast, the enhanced data by GIST separated tumor cells from the other types more clearly. Our enhancement was significantly improved by the feature extraction process, resulting in better separation and identification of cellular subpopulations. In human breast cancer, the overexpression of ERBB2 has been suggested a strong association with poor prognosis [47]. Therefore, we visualized the expression of ERBB2 in both the raw and the enhanced datasets (Fig. 3C). In the raw dataset, the expression pattern for ERBB2 appeared noisy, and differed significantly among distinct tissues. After being enhanced by GIST, the high and low expression regions of ERBB2 were more obvious, and the separation of different expression levels was significantly clearer than the previous (*t* test, *P* = 0.00084). We further illustrated the results of GIST-enhanced gene expression using violin plots (Fig. 3D). Figure 3E shows the changes in the expression of the specific gene ESR1 [48] before and after enhancement. The original expression of ESR1 was relatively low and difficult to be identified in the raw data. However, GIST enhances the identification, which is critical for tailoring hormone therapy strategies and predicting treatment response in breast cancer patients. GIST identified more differentially expressed genes (DEGs) in specific cell populations, revealing expression variations undetected in the raw data and providing deeper insights into gene expression under varying conditions.

**Fig. 4.**
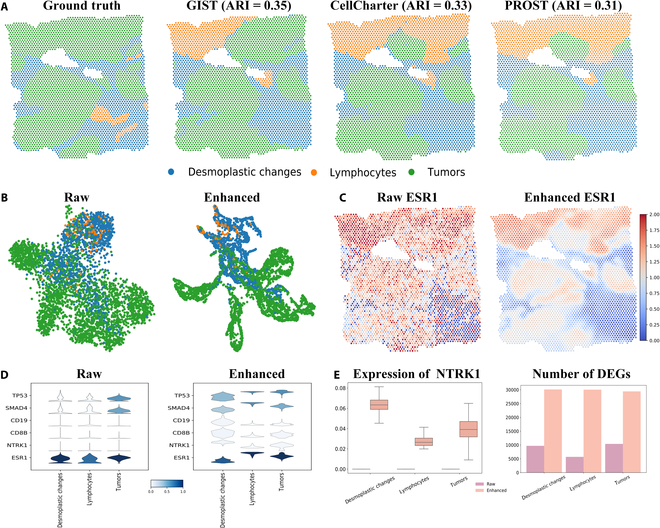
GIST improved gene expression analysis of tumors in human breast cancer (Space Ranger 1.0.0). (A) Spatial region identification comparison among GIST (ARI = 0.35), CellCharter (ARI = 0.33), and PROST (ARI = 0.31). (B) Visualization of the raw and enhanced UMAP results. In the raw data, tumor cells were scattered across various clusters, intermingling with lymphocytes and desmoplastic changes, indicating poor differentiation. GIST enhancement results showed more cohesive tumor cell clusters, which were distinctly separated from the lymphocytes and desmoplastic regions. (C) Spatial visualization of the denoised expression results of ESR1 in raw and enhanced datasets. GIST amplified the identification of ESR1 expression (*t* test, *P* = 0.036), improving the detection of its role in tumor development and hormone therapy resistance. (D) GIST enhanced the differential expression results of the breast cancer-related marker genes, revealing sharper and more distinct expression patterns. (E) The bar plots demonstrated raw (purple) and enhanced (orange) NTRK1 expression levels, alongside the number of DEGs across various cell clusters. GIST successfully enhanced the identification of NTRK1, which aids in predicting tumor malignancy and patient survival. Additional DEGs identified after GIST enhancement potentially serve as new biomarkers for breast cancer diagnosis and monitoring.

We evaluated the efficacy of GIST using the Human Breast Cancer (Space Ranger 1.0.0) Spatial Gene Expression Dataset. Figure 4A presented the spatial region as determined by ground truth, by GIST with CtransPath, by CellCharter, and by PROST. Overall, the tumor regions predicted by GIST are more accurate. Although this particular dataset has fewer classes, resulting in a slightly lower ARI index compared to other datasets, the overall accuracy of spatial region identification remains high. Concurrently, Fig. 4B illustrated the effect of GIST-enhanced tumor segmentation. ESR1 mutations as emerging clinical biomarkers in metastatic hormone receptor-positive breast cancer may help monitor disease progression and cause treatment resistance [49]. The GIST-enhanced gene expression of ESR1 (*t* test, *P* = 0.036) improved tumor cell detection (Fig. 4C). The violin plot in Fig. 4D quantitatively demonstrated the impact of GIST on enhancing marker gene expression. For each cell type, DEGs were better identified after GIST enhancement (Fig. 4E). These results demonstrat that the GIST’s ability to reduce noise and improve gene expression patterns in breast cancer datasets.

### GIST improves the expression pattern of specific genes in cell types in colorectal cancer

We also conducted further evaluation using human colorectal cancer datasets. The colorectal cancer samples were obtained from Discovery Life Sciences by 10x Genomics. The H&E images were acquired through sectioning, dewaxing, H&E staining, and imaging. The H&E images reveal that colorectal cancer with a proliferative connective tissue response, as well as infiltrating tumor areas with a large amount of tumor stroma. This dataset contains 9,080 cells and 18,085 gene data, categorized into 5 groups: desmoplastic changes, muscularis propria, tumor, tumor necrosis, and vessel.

**Fig. 5.**
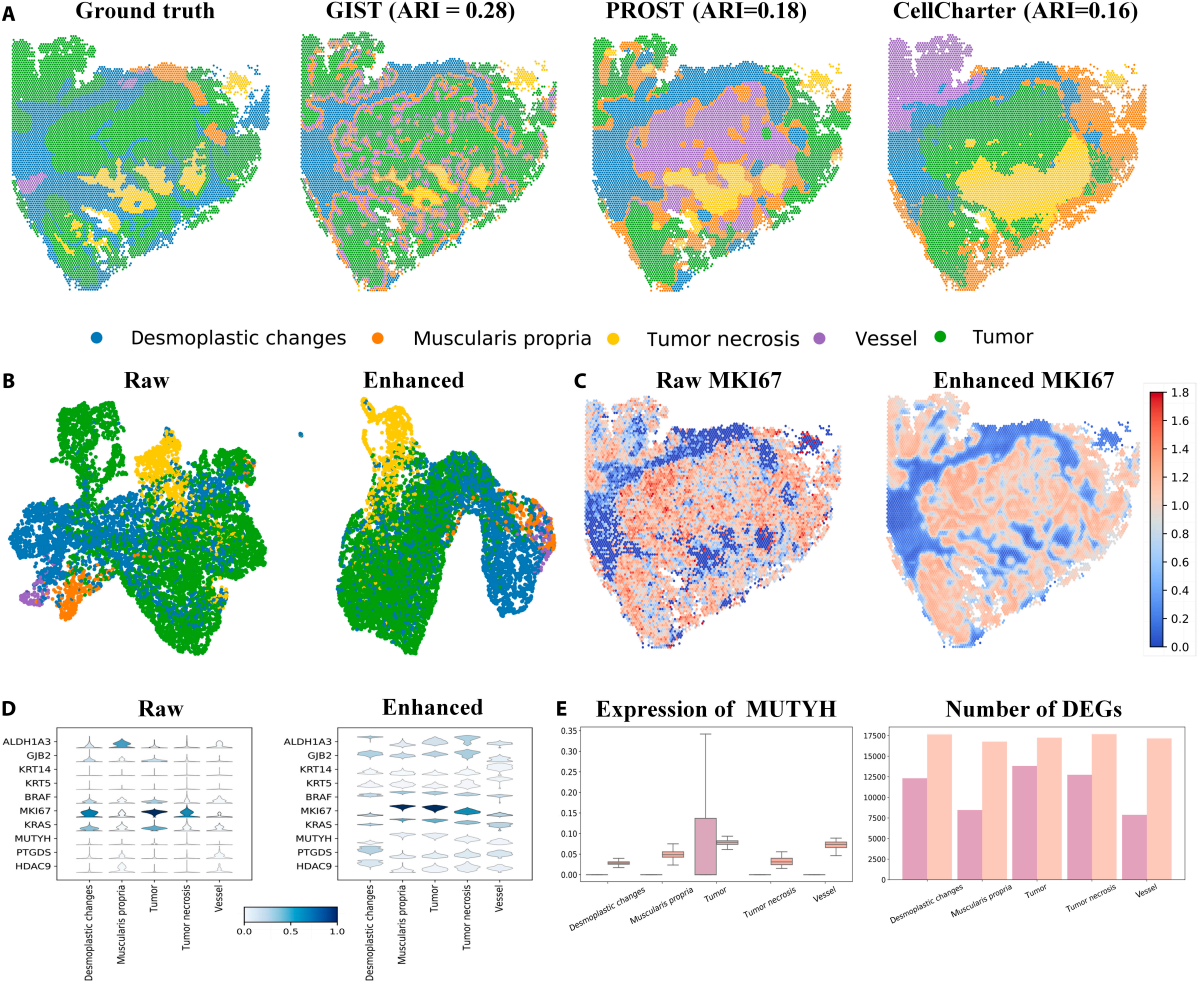
GIST improved the identification of the specific gene expression patterns in cell types associated with colorectal cancer. (A) Spatial regions predicted by GIST (ARI = 0.28) are more accurately reflect the spatial distribution of tumor and tumor necrosis regions, compared with SOTA models, such as PROST (ARI = 0.18) and CellCharter (ARI = 0.16). (B) In the raw data, cell types were not well separated, with tumor cells entangled with lymphocytes and desmoplastic changes. The enhanced clustering results by GIST showed better separate and more cohesive clusters of tumor cells. (C) Spatial visualization of MKI67 expression. The enhanced data revealed more distinct and spatially coherent regions of high MKI67 expression (red) and consistent regions of low expression (blue), successfully capturing the spatial distribution (*t* test, *P* = 0.00013). (D) Comparison of raw and enhanced marker gene expression across different cell types, particularly for tumor-associated genes like MKI67, KRAS, and BRAF. (E) As an example, the visualization of raw (purple) and enhanced (orange) MUTYH expression demonstrated how GIST helped provide clearer identification, especially in tumor regions, allowing for more accurate comparisons of DEGs across cell clusters.

Figure 5A shows the visualization of the original and the predicted spatial domains using GIST, PROST, and CellCharter. The GIST-predicted tumor (green) and necrosis (red) regions matched the ground truth with high accuracy. In the UMAP plot (Fig. 5B), GIST clustered tumor cells that were originally dispersed. Additionally, the tumor and necrosis regions were located near each other. MKI67 is a potential diagnostic and prognostic biomarker in microsatellite instability stage II/III high colorectal cancer [50]. The enhanced visualization of MKI67 gene expression showed significantly (*t* test, *P* = 0.00013) clearer areas of high MKI67 expression with greater spatial continuity, indicating that the enhancement effectively captured the spatial pattern of MKI67 expression. Similarly, regions with low MKI67 expression appeared to be more uniform in the enhanced visualization (Fig. 5C). Additional downstream tasks, visualized using violin plots (Fig. 5D) for the detection of DEGs (Fig. 5E), suggested that GIST improved the interpretation of gene expression patterns by effectively denoising the gene expression data before integrating it with staining images.

## Discussion

Spatial transcriptomic techniques consistently provide high-resolution tissue histology images. While histology examination remains the gold standard for cancer diagnostics and disease understanding due to its rich cell morphology information, current methods for processing ST data have not fully utilize this morphological information, mostly relying instead on it solely for localization or comparing basic cellular similarity. In contrast, the field of digital pathology has rapidly advanced, with deep learning-enabled microscopic image analysis showing promising applications in computer-aided diagnostics. Recent developments in histopathology image foundation models have enabled accurate extraction of tissue-level cellular image features. In this work, we present GIST, a novel approach that leverages pretrained self-supervised histology image foundation models to extract features and employs a hybrid graph transformer to efficiently fuse these image features with transcriptomic features. In our experimental setup, we used multiple state-of-the-art pretrained histology foundation models as backbones, including CTransPath, Virchow2, Gigapath, and UNI. All models were trained on millions of diagnostic H&E-stained images and used for extracting cellular morphological features. Despite differences in their architectures and pretraining datasets, these backbones efficiently capture both local and global cellular features from processed patches for aiding downstream histopathology diagnostic tasks. CTransPath employs contrastive learning to generate and refine feature vectors via parallel network processing, contrastive learning, and exponential moving average (EMA). In contrast, Virchow2, Gigapath, and UNI use a self-supervised student–teacher network framework, where the student progressively learns to extract meaningful features under the guidance of the teacher network. These complementary strengths shaped the selection of two foundation model families for our study.

The performances from different backbone variants in the framework were introduced in Table S1, including GIST with UNI, GIST with CTransPath, GIST with Virchow2, and GIST with Gigapath for processing SCST data. Among these, GIST with UNI demonstrated superior overall performance in the lung cancer dataset, achieving a consistent score of 0.64 across the lung5-1, lung5-2, and lung5-3 datasets. In contrast, GIST with CTransPath excelled in both the breast cancer and colorectal cancer datasets, showcasing its superior adaptability to these data types. Overall, models based on the GIST framework demonstrate notable improvements in the accuracy and reliability of spatial transcriptome analyses. In the main text, we present the results of the GIST with UNI model for visualization and detailed analysis in the lung cancer dataset. For the breast and colorectal cancer datasets, the results of the GIST with CTransPath model overperformed the baseline models and showed the advantages of pathology-informed approaches in enhancing spatial transcriptome analyses.

GIST employed a multimodal strategy to fuse histological image with spatial transcriptomic features, effectively leveraging the strengths of both modalities. By integrating the local and global context from staining images with spatial transcriptome data at the cellular level, GIST enhanced precision in distinguishing cellular structures. GIST's hybrid graph transformer efficiently addressed the challenge of dropout events, where certain genes may remain undetected despite active expression. This integration enhances gene expression data by mitigating dropouts and denoising, enabling better domain segmentation and biomarker identification, even with incomplete data.

The identification of genes and spatial domains are 2 essential tasks in SCST data analysis for understanding tissue architecture and disease microenvironments using spatial transcriptomic data [51]. In this study, we focused on assessing GIST’s generalizability in these 2 tasks. Although these tasks target to learn different information (domain recognition focuses on classifying cell types across an image, while gene identification focuses on identifying and classifying specific genes), both require models capable of extracting meaningful insights from complex biomedical images. In our experiments, we visualized and compared spatial domain detection and clustering results before and after GIST enhancement. We found that GIST was more accurate in delineating structures with different anatomical contours. Moreover, we demonstrated the efficacy of GIST in identifying differentially expressed marker genes by visualizing their enhanced expression in spatial maps. 

In addition to GIST’s usability in tumor-related tasks, we also explored its usage in recognizing noncancerous tissue structures. Our analysis of the dorsolateral prefrontal cortex (DLPFC) dataset showed GIST’s acceptable accuracy in brain region recognition (Figs. S2 to S4). However, because all of these histology image foundation models were predominantly developed using large-scale datasets of neoplastic histopathology images, these models often fail to encode noncancerous phenotypes hindering accurate spatial mapping of gene expression in human cerebral cortex. Brain-related datasets are relatively scarce compared to other fields, given the challenges in obtaining brain tissue samples and the smaller dataset sizes. 

Apart from GIST's superior performance and technical advantages, GIST can be further improved in the future. First, current histology foundation models are not yet single-cell specific when encoding morphological features. Further improvements, such as better cell segmentation, could be evaluated [52]. Our investigation also did not explicitly explore the potential of integrating foundation models in transcriptomics or genomics, which may require more computational resources for pretraining but extract more biologically meaningful features from the gene expression data [53]. Additionally, given the broad scope of the assessment, our focus has been primarily on publicly available cancer datasets, with limited consideration of other diseases in anatomical pathology or normal tissue structures. Future work will aim to further evaluate the model’s generalizability to other diseases, such as neurological disorders in anatomical pathology [20].

## Methods

### Data preprocessing for gene expression and histology images

SCST contains multimodal features including gene expression, spatial location, and histological information [54]. We obtained gene expression profiles and gene location matrices from the dataset. We employed the function of var_names_make_unique from the Scanpy library to ensure the consistency of the variable names. Then, we normalized the gene expression profiles to ensure a consistent data scale across cells, mitigating the impact of sample size differences in subsequent analyses. The logarithm was applied to each data element to approximate a normal distribution. For image processing, a systematic procedure was adopted for various datasets. Each cell was cropped into a patch with 3 × 240 × 240 pixels, with the cell positioned at the center. Spatial coordinates were used to identify neighboring cells within a Euclidean distance of ≤80 pixels, constructing a spatial neighborhood map and ensuring consistency in preprocessing across datasets.

### Feature extraction

We employed both contrastive learning-based and knowledge distillation-based histology image foundation models to extract image features. The contrastive learning-based image feature extractor (i.e., CTransPath), two views, $x_{1}$ and $x_{2}$, were created from the original histology image patch *x*. These views, along with the original image, were fed into 3 parallel branches of CTransPath backbone, with the branches for $x_{1}$ and $x_{2}$ sharing the same model parameters. Stemmed from MoCo v3 [55], $x_{1}$ was processed by an online network’s backbone, while $x_{2}$ was passed through a target network for extracting feature vectors $z_{1}$ and $z_{2}$. To increase the number of positive samples, the original image *x* was also processed through an additional branch to generate a feature vector *z*. The target branch and the original image branch queried the memory bank for getting semantically similar samples. These similar samples within the memory bank were ranked by their similarity, with the top *S* samples designated as positives and the reminding ones as negatives. During contrastive learning, the feature vector $z_{1}$ from the online branch served as an anchor, pulling the positive samples closer and pushing the negative samples further away from itself in the embedding space.

 CTransPath replaced the patch partition of Swin Transformer with a nonlinear mapper based on CNN, which improved the stability of network training and captured more local features of the image. The adjusted feature extractor scanned local information from histology images using the CNNmodule and utilized the transformer module to obtain global features of histology images (Fig. S1C). In each image, each patch was sampled centered as a cell-of-interest in H×W×3. The image patch of H×W×3 was fed into the CNN module to obtain the local feature map $F\in {\mathbb{R}}^{\frac{H}{4}\times \frac{W}{4}\times C}$. The CNN module were with 3 consecutive convolution layers with kernel sizes 3×3, 3×3, and 1×1. Convolution kernels of different sizes capture features of different scales in the image patch, i.e., local features. The local feature map *F* was then input into the Swin Transformer, and hierarchical features were obtained through 4 Swin Transformer blocks. In each layer of the Swin Transformer (Fig. S1C), the input feature was downsampled. The Swin Transformer consists of a window-based multi-head self-attention (W-MSA) layer and a shift-window-based multi-head self-attention (SW-MSA) layer. The Swin Transformer [39] has 2 GELU nonlinear multilayer perceptrons (MLPs) in between, along with a LayerNorm (LN) layer in between each MSA module and MLP module, while each module is connected with residuals. The continuous Swin Transformer is calculated as follows:

$$\begin{array}{cc} & \;{\hat{z}}^{l}=W-MSA\left(LN\left(z^{l-1}\right)\right)+z^{l-1},\\ & \;z^{l}=MLP\left(LN\left({\hat{z}}^{l}\right)\right)+{\hat{z}}^{l},\\ & {\hat{z}}^{l+1}=SW-MSA\left(LN\left(z^{l}\right)\right)+z^{l},\\ & z^{l+1}=MLP\left(LN\left({\hat{z}}^{l+1}\right)\right)+{\hat{z}}^{l+1} \end{array}$$ (1)

where ${\hat{z}}^{l}$ and ${\hat{z}}^{l+1}$ represent the product of the (S)W-MSA module and the MLP module for block *l*.

For knowledge distillation-based image feature extractor, we chose a family (i.e., UNI, Virchow2, and Gigapath) utilizing DINOv2 [56], a self-supervised framework based on the teacher–student network architecture. The patches from the original image were augmented to create 2 views, $x_{1}$ and $x_{2}$. These augmented views were then fed into the student network. The parameters of the teacher network were updated using an EMA of the student network’s parameters. The knowledge-distillation-based image feature extractor also employed the augmented view of the original image as a random mask. The unmasked portion of these augmented views was used in the teacher’s network, while the masked portion was used in the student network. In addition, alignment losses were used for assessing the consistency between feature representations generated by a network of students and teachers. 

### Hybrid graph transformer model

Hybrid graph transformer model contained 3 major modules: the construction of cellular spatial graph, graph transformer model, and loss function. In the construction phase of single-cell spatial graph, we obtained gene expression profiles and position matrices of cells from spatial transcriptome data. In single-cell spatial graph, each graph node $v_{i}$ represents a cell, and the edges between nodes represent 2 cells that are neighbours of each other. The adjacency was measured by Euclidean distance. Specifically, each cell includes gene expression data from gene expression profiles and an N×N image patch centered on a cell-of-interest.

The graph transformer model contains 3 graph transformer autoencoders (Fig. S1B) for different usages, including image autoencoder, transcriptome autoencoder, and hybrid autoencoder. The image autoencoder took the features $z_{M,i}$. Similarly, the transcriptome autoencoder utilized the raw cell expression features $z_{g,i}$. Then, these two features were concatenated as the hybrid features and fed into the hybrid autoencoder to obtain hybrid embedding features $z_{h,i}$. In order to better maintain the spatial information of the data, the features of the neighbouring cells were also fed into the graph transformer model. The implementation was done by single-cell spatial graph. A mean squared error (MSE) self-supervised loss was applied to guide the model in learning how to integrate image embedding features, gene embedding features, and hybrid embedding features. The overall loss function is defined as follows:

$$L=\sum_{i=1}^{N}\lambda_{1}L_{M,i}+\lambda_{2}L_{g,i}+L_{h,i}$$ (2)

where *N* is the total number of cells and $L_{M,i}$ is the gene embedding loss. $L_{g,i}$ is the image embedding loss, and $L_{h,i}$ is the hybrid embedding loss. In constructing the overall loss function, $\lambda_{1}$ and $\lambda_{2}$ hyperparameters are also used to balance the weights of different loss components, which should satisfy $\lambda_{1}$, $\lambda_{2}$ ≥ 0.

### The state-of-the-art models for baseline comparisons

To benchmark spatial domain segmentation performance, we compared GIST against nine state-of-the-art methods, each developed using different model architectures and technical focuses, including STAGATE [14], spaGCN [13], BayesSpace [12], Scanpy [10], Seurat [9], stLearn [11], SiGra [30], PROST [17], and CellCharter [18]. STAGATE, SpaGCN, and SiGra utilize graph-based methods to capture cell spatial correlations and uncover spatial domains. BayesSpace employs a comprehensive Bayesian framework to enhance clustering resolutions. PROST uses unsupervised clustering to identify spatial domains and quantitatively assess spatial variations in gene expression patterns. CellCharter excels in identifying biologically meaningful cell niches. Additionally, Scanpy is a tool specialized in single-cell gene expression analysis and managing annotated transcriptomic data. stLearn maps cell progression within tissues and explores regions exhibiting significant intercellular interactions.

### Comparison measurement and experiment parameters

The ARI is a metric for measuring the similarity between 2 data partitions and is commonly used to evaluate the performance of clustering algorithms in ST data analysis. To compare the similarity between domain segmentation results, we employed the ARI to measure the similarity to assess the accuracy and reliability of each method. The formula for the ARI is defined as below:

$$\begin{array}{c} \begin{array}{c} \begin{array}{c} \mathrm{ARI}=\frac{\sum_{\mathrm{ij}}\left(\begin{array}{c} n_{\mathrm{ij}}\\ 2 \end{array}\right)-\left[\sum_{i}\left(\begin{array}{c} a_{i}\\ 2 \end{array}\right)\sum_{j}\left(\begin{array}{c} b_{j}\\ 2 \end{array}\right)/\left(\begin{array}{c} n\\ 2 \end{array}\right)\right]}{\frac{1}{2}\left[\sum_{i}\left(\begin{array}{c} a_{i}\\ 2 \end{array}\right)+\sum_{j}\left(\begin{array}{c} b_{j}\\ 2 \end{array}\right)\right]-\left[\sum_{i}\left(\begin{array}{c} a_{i}\\ 2 \end{array}\right)\sum_{j}\left(\begin{array}{c} b_{j}\\ 2 \end{array}\right)/\left(\begin{array}{c} n\\ 2 \end{array}\right)\right]} \end{array} \end{array} \end{array}$$ (3)

where *n* is the total number of samples, $n_{i,j}$ is the number of sample pairs belonging to both $A_{i}$ and $B_{j}$. $a_{i}$ is the number of samples in $A_{i}$, and $b_{j}$ is the number of samples in $B_{j}$. The value of ARI usually ranges from −1 to 1, where 1 means that the 2 parts are in perfect agreement, 0 means that the agreement is equivalent to random assignment, and −1 means that 2 parts are completely against each other.

We optimized several parameters to enhance experimental performance, employing a grid-search approach informed by insights from previous studies [30]. During image feature extraction, we determined the optimal patch size referring to the previous empirical results from SiGra. The comparison included 3 configurations: a size of 3 × 120 × 120 as used by SiGra, a size of 3 × 240 × 240 augmented by SiGra, and a standard size of 3 × 224 × 224 commonly used in histology image foundation models. Our experiments suggest that the size of 3 × 240 × 240 pixels reached the best performance. During model training, we utilized a learning rate of 0.001, 1,000 epochs, a maximum gradient norm of 5, and the Adam optimizer with a weight decay of 0.0001. The loss weight on both gene and image features was set to 0.1. To construct spatial networks among cells, A Euclidean distance threshold was adjusted for each dataset to ensure comparable average neighbor counts across spatial maps. Specifically, we set the Euclidean distance to 80 for lung cancer, 300 for breast cancer, and 20 for colorectal cancer.

## Data Availability

The NanoString CosMx SMI data are available at https://nanostring.com/products/cosmx-spatial-molecular-imager/ffpe-dataset/nsclc-ffpe-dataset/. This dataset has 8 sections, each with 20 to 45 FOVs. 10x Visium dataset of human breast cancer can be downloaded at https://www.10xgenomics.com/datasets/human-breast-cancer-ductal-carcinoma-in-situ-invasive-carcinoma-ffpe-1-standard-1-3-0 and https://www.10xgenomics.com/datasets/human-breast-cancer-block-a-section-1-1-standard-1-0-0. This dataset was derived from 10x Genomics on FFPE human breast tissue sourced from BioIVT’s Asterand Human Tissue Repository. 10x Visium dataset of human colorectal cancer can be downloaded at https://www.10xgenomics.com/datasets/human-colorectal-cancer-11-mm-capture-area-ffpe-2-standard. The python implementation of GIST is publicly available at https://github.com/lengjk1214/GIST.
