# Supplementary material for: Deep Learning-Enabled Integration of Histology and Transcriptomics for Tissue Spatial Profile Analysis
Source: Research (Wash D C). 2025 Jan 17;8:0568. doi: 10.34133/research.0568 (PMC11739434; doi:10.34133/research.0568)
Supplement: Supplementary 1 — Tables S1 and S2 Figs. S1 to S6 [file research.0568.f1.pdf]

SUPPLEMENTARY INFORMATION

GIST: Gene expression and histology Integration for SpaTial cellular profiling

Table of Contents

Supplementary Table. 1: Comparison experiments of GIST(UNI), GIST(CtransPath), GIST(Virchow2), GIST(Gigapath) and MUSE were conducted using datasets from lung cancer, breast cancer, and colorectal cancer. . . . 2

Supplementary Table. 2: Comparison experiments of GIST(UNI), CellCharter, SiGra, MUSE were conducted using datasets from lung cancer, breast cancer, and colorectal cancer. . . . 2

Supplementary Fig. 1: Boxplot of the ARI scores of nine methods in all the 8 lung cancer slices. Illustration of the multi-head transformer layer and CNN-Augmented Swin Transformer. . . . 3

Supplementary Fig. 2: Visualization and evaluation of spatial domains identified by GroundTruth, SiGra and GIST in DLPFC 151507, 151508, 151509, 151510 tissue slices. . . . 4

Supplementary Fig. 3: Visualization and evaluation of spatial domains identified by GroundTruth, SiGra and GIST in DLPFC 151669, 151670, 151671, 151672 tissue slices. . . . 5

Supplementary Fig. 4: Visualization and evaluation of spatial domains identified by GroundTruth, SiGra and GIST in DLPFC 151673, 151674, 151675, 151676 tissue slices. . . . 6

Supplementary Fig. 5: Visualization and evaluation of spatial domains identified by GIST, SiGra, and CellCharter in lung5-1, lung5-2, lung5-3, and lung6 tissue slices. . . . 7

Supplementary Fig. 6: Visualization and evaluation of spatial domains identified by GIST, SiGra, and CellCharter in lung9-1, lung9-2, lung12, and lung13 slices. . . . 8

Supplemental Table 1: Comparison experiments of GIST(UNI), GIST(CtransPath), GIST(Virchow2) and GIST(Gigapath) were conducted using datasets from lung cancer, breast cancer, and colorectal cancer.

**Datasets**		**GIST (UNI[1])**	**GIST (CtransPath[2])**	**GIST (Virchow2[3])**	**GIST (Gigapath[4])**
**Lung cancer**	**lung5-1**	0.64	0.6	0.63	0.6
	**lung5-2**	0.64	0.66	0.66	0.66
	**lung5-3**	0.64	0.61	0.64	0.62
	**lung6**	0.37	0.29	0.26	0.24
	**lung9-1**	0.59	0.62	0.57	0.6
	**lung9-2**	0.44	0.39	0.43	0.41
	**lung12**	0.18	0.15	0.18	0.16
	**lung13**	0.62	0.56	0.61	0.6
**Breast cancer**	**Breast cancer1**	0.52	0.61	0.54	0.42
	**Breast cancer2**	0.35	0.35	0.36	0.31
**Colorectal cancer**	**Colorectal**	0.25	0.28	0.22	0.23

Supplemental Table 2: Comparison experiments of GIST(UNI), CellCharter, SiGra, PROST and MUSE were conducted using datasets from lung cancer, breast cancer, and colorectal cancer.

**Datasets**		**GIST (UNI[1])**	**CellCharter[5]**	**SiGra[6]**	**PROST[7]**	**MUSE[8]**
**Lung cancer**	**lung5-1**	0.64	0.66	0.59	0.04	0.01
	**lung5-2**	0.64	0.57	0.5	0.04	0.02
	**lung5-3**	0.64	0.65	0.51	0.04	0.01
	**lung6**	0.37	0.18	0.27	0.03	0.03
	**lung9-1**	0.59	0.52	0.55	0.06	0.04
	**lung9-2**	0.44	0.19	0.42	0.06	0.02
	**lung12**	0.18	0.32	0.11	0.03	0.01
	**lung13**	0.62	0.5	0.45	0.07	0.11
**Breast cancer**	**Breast cancer1**	0.52	0.4	0.01	0.21	0.24
	**Breast cancer2**	0.35	0.33	0.04	0.31	0.20
**Colorectal cancer**	**Colorectal**	0.25	0.16	0.13	0.18	0.11

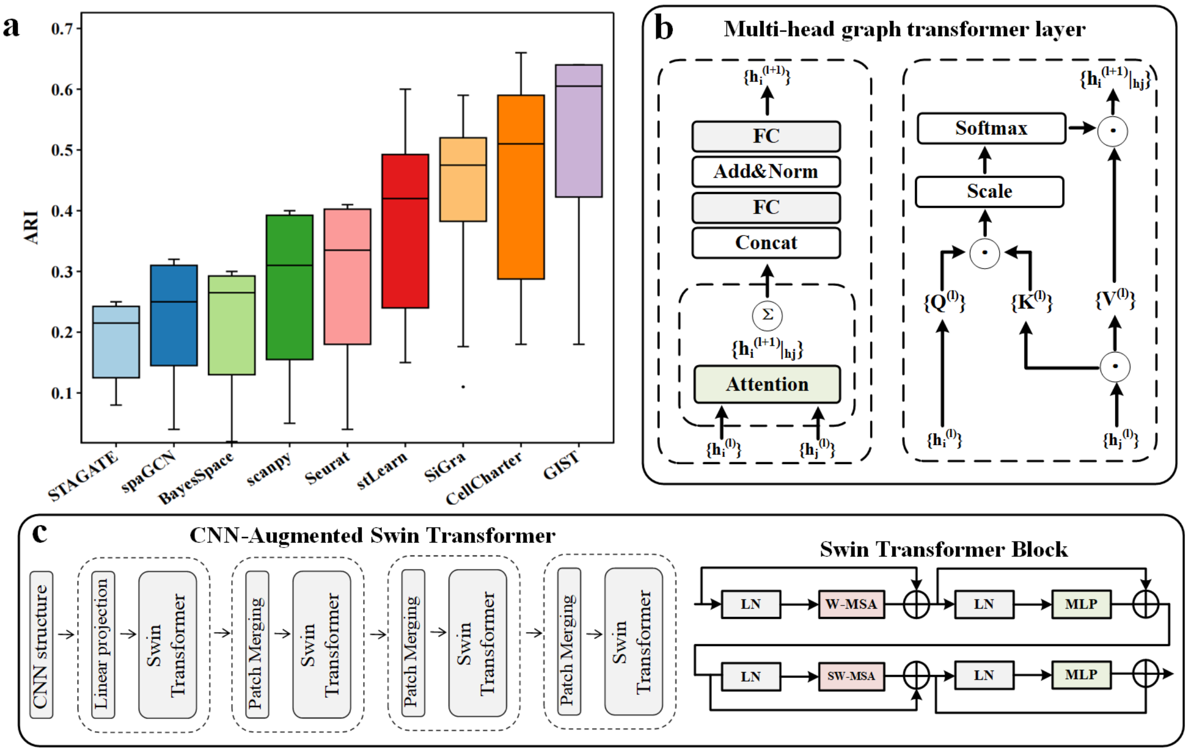

Supplementary Figure 1. **a** Boxplot of the ARI scores of nine methods in all the 8 lung cancer slices. The GIST achieves median ARI of 0.61, significantly outperforming other methods. CellCharter follows with an ARI of 0.50, then SiGra at 0.48, stlearn at 0.42, seurat at 0.34, scanpy at 0.31, BayesSpace at 0.27, spaGCN at 0.25 and STAGATE at 0.22. **b** The overall architecture of a multi-head transformer layer. Multi-head transformer layer contains two modules, a graph transformer layer and an attention module. **c** The structure of CNN-Augmented Swin Transformer and Swin Transformer Block. The image patch is fed into the CNN architecture which segments the input image into non-overlapping patches. To generate a hierarchical feature representation, histology images are sequentially fed into four different stages of the Swin Transformer block for feature transformation. Swin Transformer Block includes a window-based multi-head self-attention (W-MSA) layer and a shift-window-based multi-head self-attention (SW-MSA) layer.

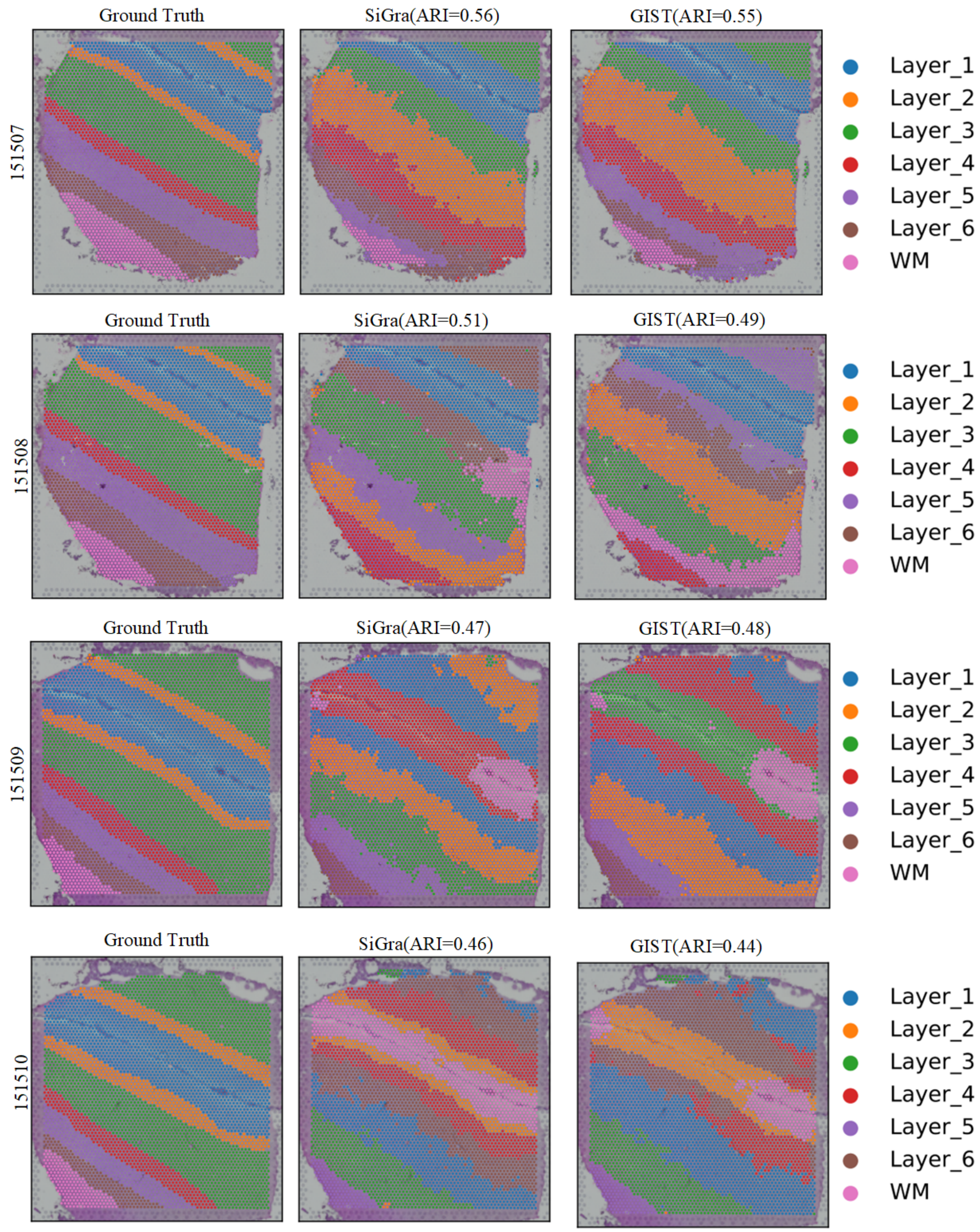

Supplementary Figure 2. Visualization and evaluation of spatial domains identified by GroundTruth, SiGra and GIST in DLPFC 151507, 151508, 151509, 151510 tissue slices. GIST demonstrated superior spatial domain recognition on the 151509 slice. Overall, GIST's domain segmentation results closely resemble those of SiGra and GroundTruth.

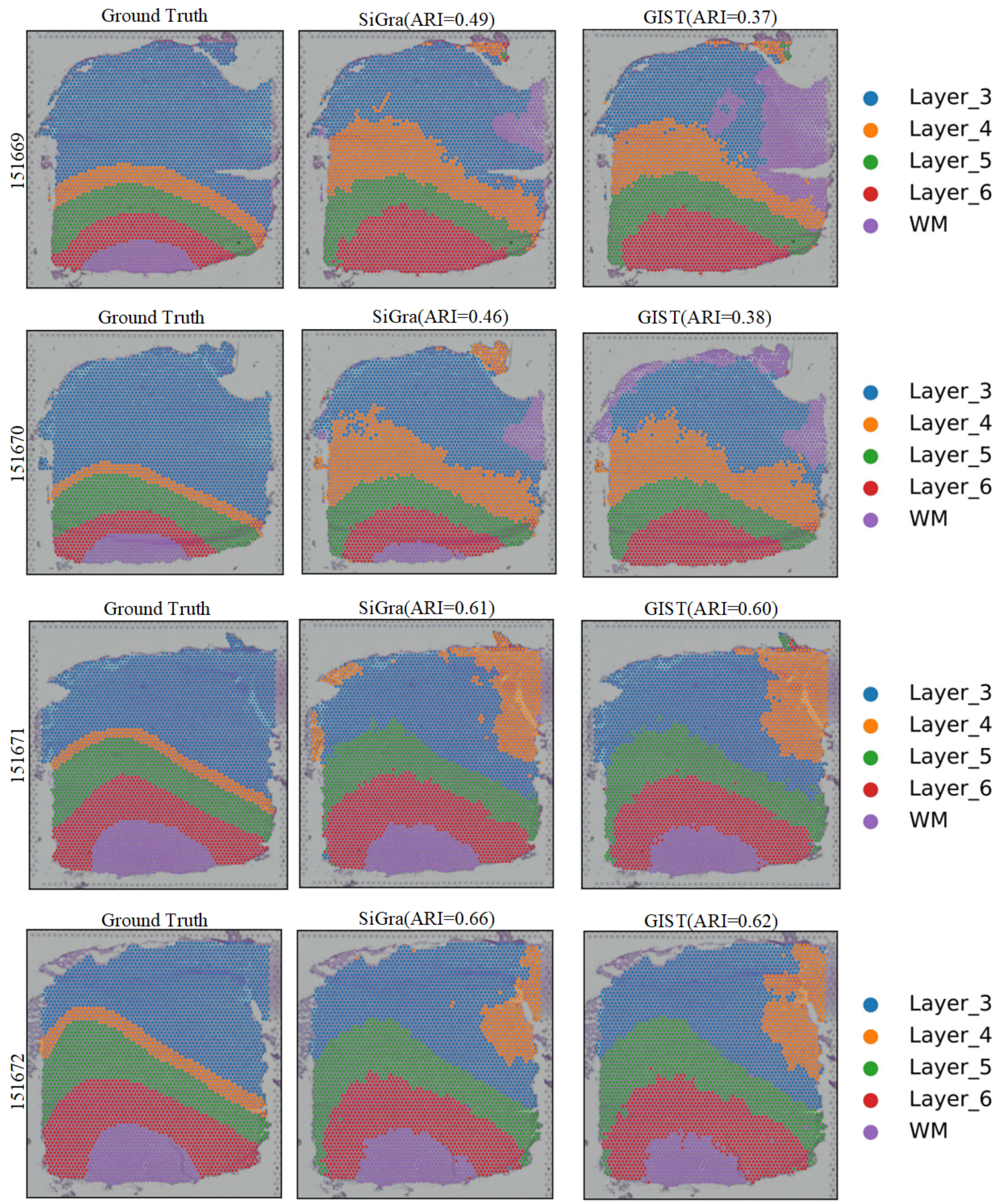

Supplementary Figure 3. Visualization and evaluation of spatial domains identified by GroundTruth, SiGra and GIST in DLPFC 151669, 151670, 151671, 151672 tissue slices. GIST's domain segmentation results closely resemble those of SiGra and GroundTruth.

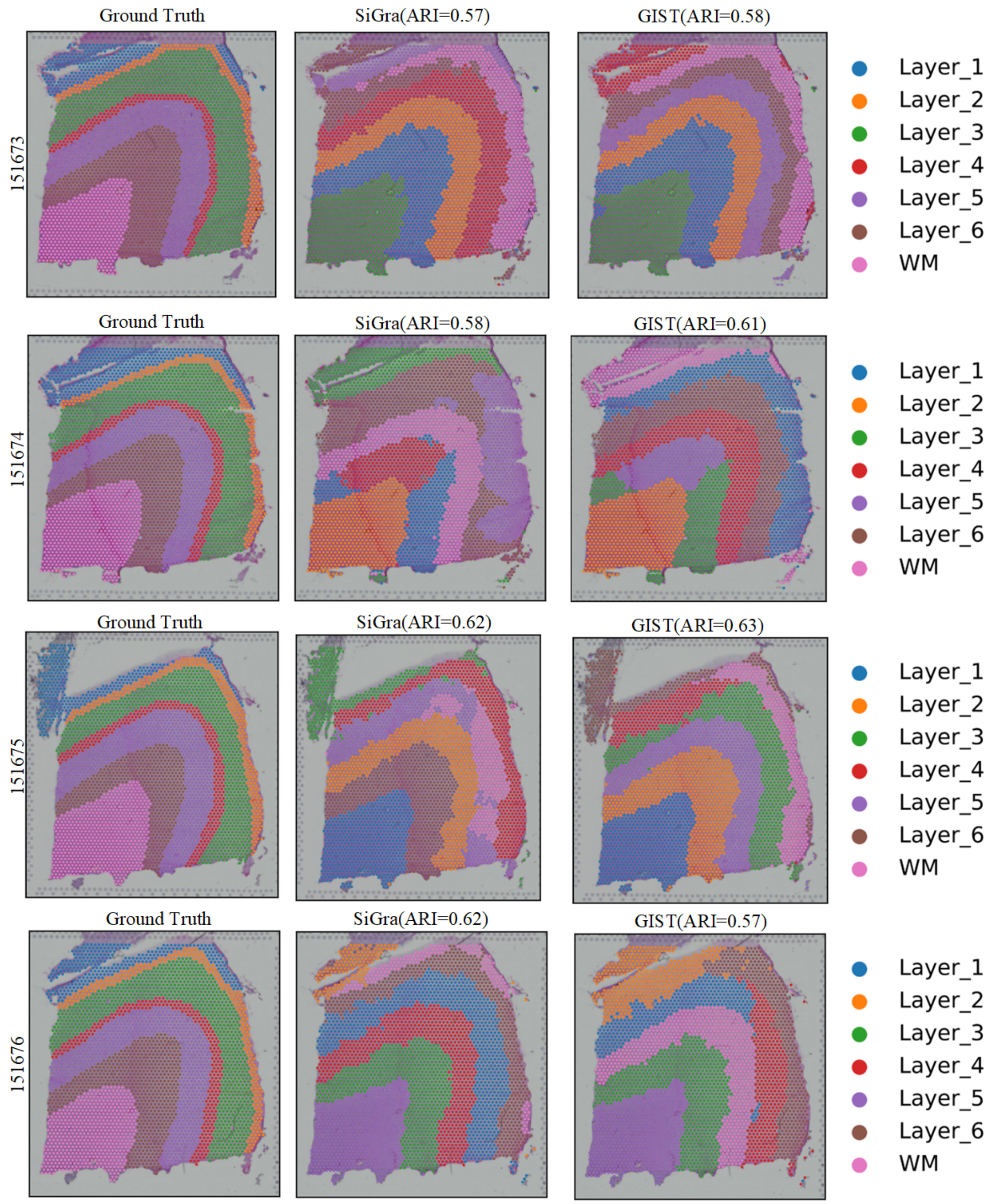

Supplementary Figure 4. Visualization and evaluation of spatial domains identified by GroundTruth, SiGra and GIST in DLPFC 151673, 151674, 151675, 151676 tissue slices. GIST shows improved spatial domain recognition on slices 151673, 151674, and 151675, with particularly strong performance on slice 151674.

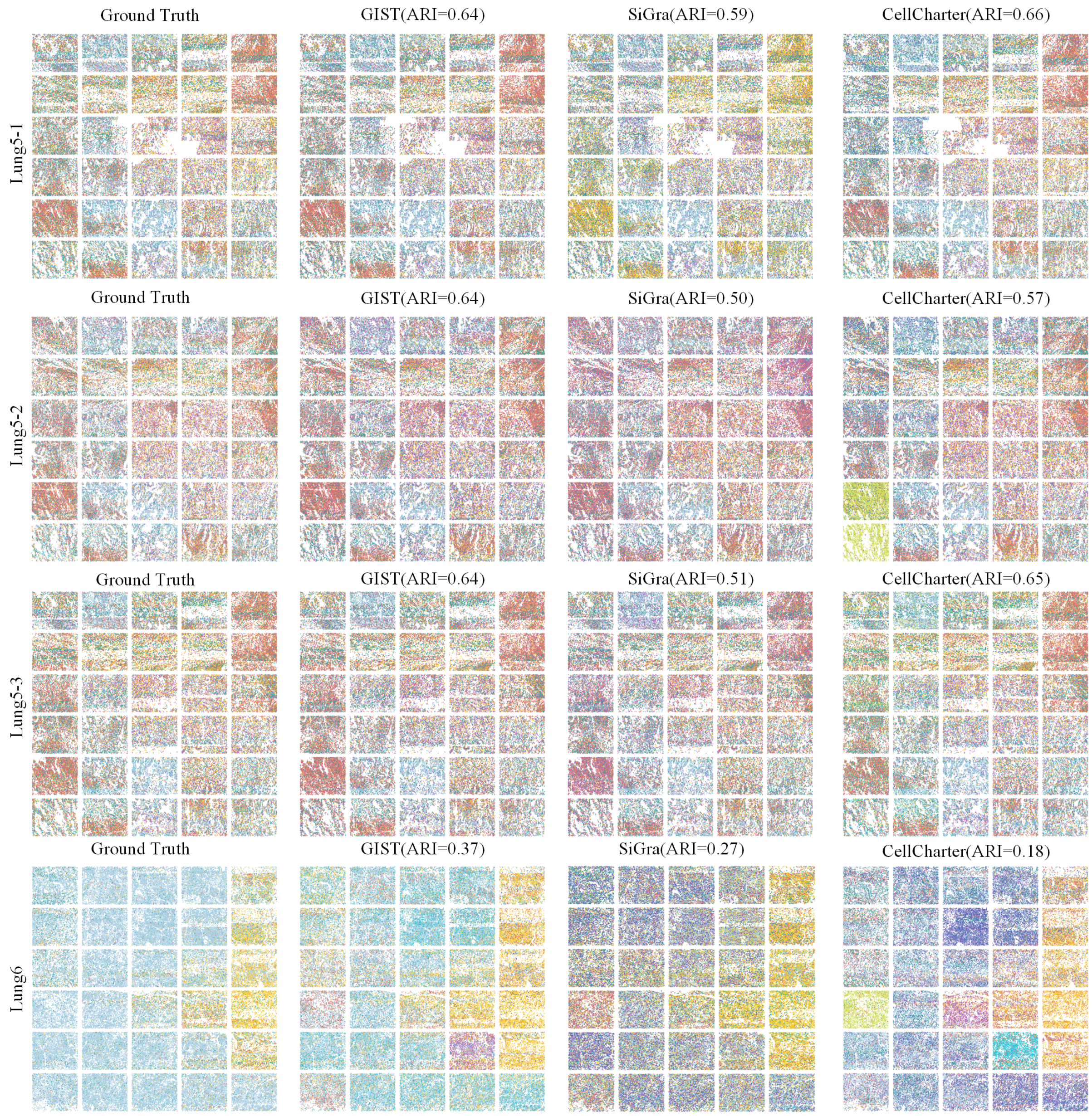

Supplementary Figure 5. Visualization and evaluation of spatial domains identified by GIST, SiGra, and CellCharter in lung5-1, lung5-2, lung5-3, and lung6 tissue slices. On the lung5-2 slice, GIST (ARI = 0.64) demonstrated superior spatial domain identification capabilities. Even though distinguishing spatial domains in lung6 slices is generally challenging, GIST (ARI = 0.37) maintained more consistent performance compared to other techniques. While GIST's ARI were marginally under those of CellCharter for the lung5-1 and lung5-3 slice, it managed to secure scores that were nearly equivalent (both at 0.64).

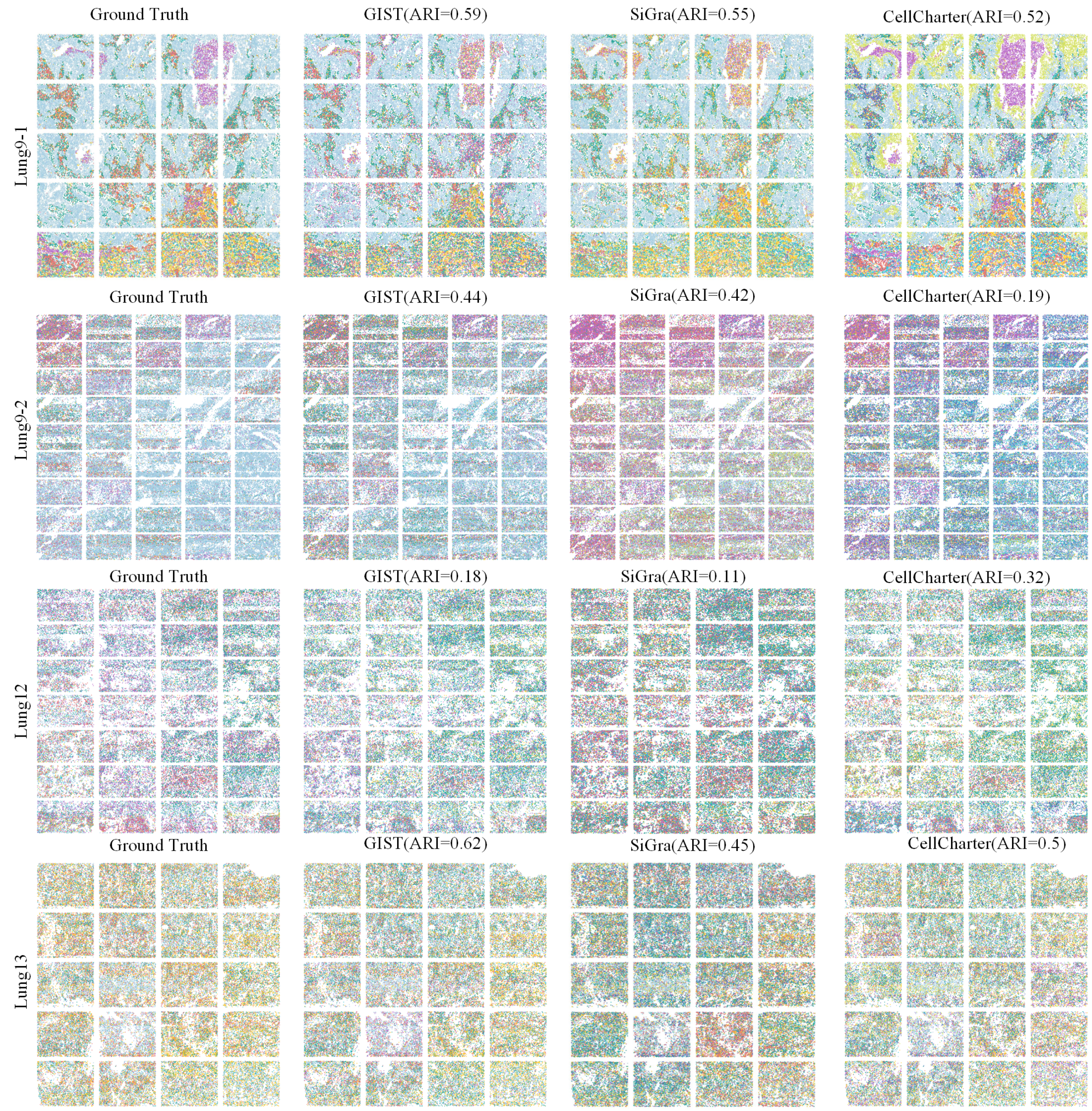

Supplementary Figure 6. Visualization and evaluation of spatial domains identified by GIST, SiGra, and CellCharter in lung9-1, lung9-2, lung12, and lung13 slices. GIST demonstrated better spatial domain recognition on lung9-1, lung9-2, and lung13 slices, and especially outperformed on lung13 slices. On Lung12 slices, despite its weaker performance, GIST still outperforms the SiGra method in terms of overall stability and is far superior to CellCharter in most cases.

Supplementary References

[1] Chen R J, Ding T, Lu M Y, et al. Towards a general-purpose foundation model for computational pathology[J]. *Nature Medicine*, 2024, 30(3): 850-862.

[2] Wang X, Yang S, Zhang J, et al. Transformer-based unsupervised contrastive learning for histopathological image classification[J]. *Medical image analysis*, 2022, 81: 102559.

[3] Zimmermann E, Vorontsov E, Viret J, et al. Virchow2: Scaling Self-Supervised Mixed Magnification Models in Pathology[J]. *arXiv preprint* arXiv:2408.00738, 2024.

[4] Xu H, Usuyama N, Bagga J, et al. A whole-slide foundation model for digital pathology from real-world data[J]. *Nature*, 2024: 1-8.

[5] Varrone M, Tavernari D, Santamaria-Martínez A, et al. CellCharter reveals spatial cell niches associated with tissue remodeling and cell plasticity[J]. *Nature Genetics*, 2024, 56(1): 74-84.

[6] Tang Z, Li Z, Hou T, et al. SiGra: single-cell spatial elucidation through an image-augmented graph transformer[J]. *Nature Communications*, 2023, 14(1): 5618.

[7] Liang Y, Shi G, Cai R, et al. PROST: quantitative identification of spatially variable genes and domain detection in spatial transcriptomics[J]. *Nature Communications*, 2024, 15(1): 600.

[8] Bao F, Deng Y, Wan S, et al. Integrative spatial analysis of cell morphologies and transcriptional states with MUSE[J]. *Nature biotechnology*, 2022, 40(8): 1200-1209.
